# Constrictive pericarditis caused by viral myocarditis in children: a brief report

**DOI:** 10.1186/s13019-022-01969-6

**Published:** 2022-08-28

**Authors:** Haiyan Lei, Dandan Wang, Haiyong Wang, Yuansheng Xu

**Affiliations:** 1 Department of Functional Examination in Children, Lanzhou University Second Hospital, Lanzhou, China; 2 The Department of Ultrasound Medicine, The First Affiliated Hospital, Xi’an Jiaotong University, Xi’an, Shanxi Province China; 3 Department of Cardiology, Lanzhou University Second Hospital, Lanzhou, China

**Keywords:** Heart, Ultrasonic, Coxsackie virus

## Abstract

**Background:**

Constrictive pericarditis is more common in adults and has a low incidence in children. Here is a case of coxsackie virus induced myocarditis in children, resulting in constrictive pericarditis.

**Conclusion:**

Chronic inflammation of viral myocarditis can cause inflammatory changes of pericarditis and cause constrictive pericarditis.

## Background

Chronic inflammation of viral myocarditis is one of the causes of constrictive pericarditis in children.

## Aims

A case of constrictive pericarditis due to viral myocarditis was diagnosed and treated surgically.

## Method

A 12-year-old boy was diagnosed with viral myocarditis in November 2018. The patient was readmitted in August 2020 due to “intermittent cough with abdominal distension for two weeks”, Physical examination showed normal development, moderate nutrition, poor spirit, a systolic “blow-like” murmur in the precardiac area, and a positive shift dullness. The liver was 5 cm subcostal and 6 cm subxiphoid, strong quality, pitting edema of both lower limbs, no other obvious abnormalities.

**Fig. 1:**
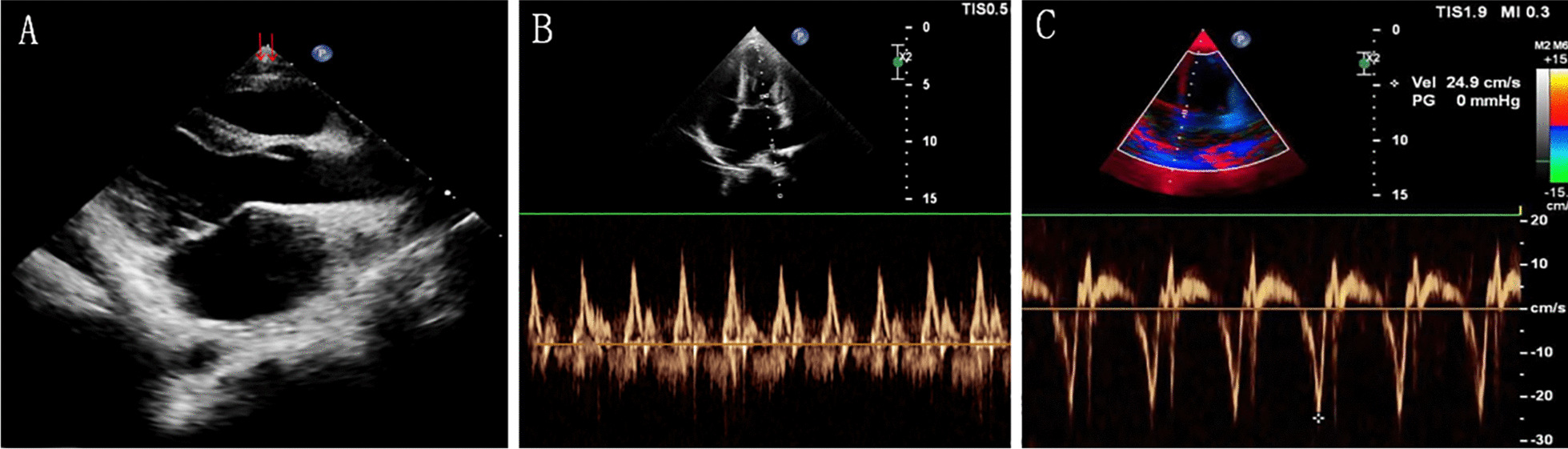
**A** Pericardial thickening in the long axis of the apex, anterior to the right ventricle (red arrow). **B** The flow velocity of the mitral valve increased with the variation of respiration. **C** Tissue Doppler showed no abnormal atrioventricular annulus velocity

**Fig. 2:**
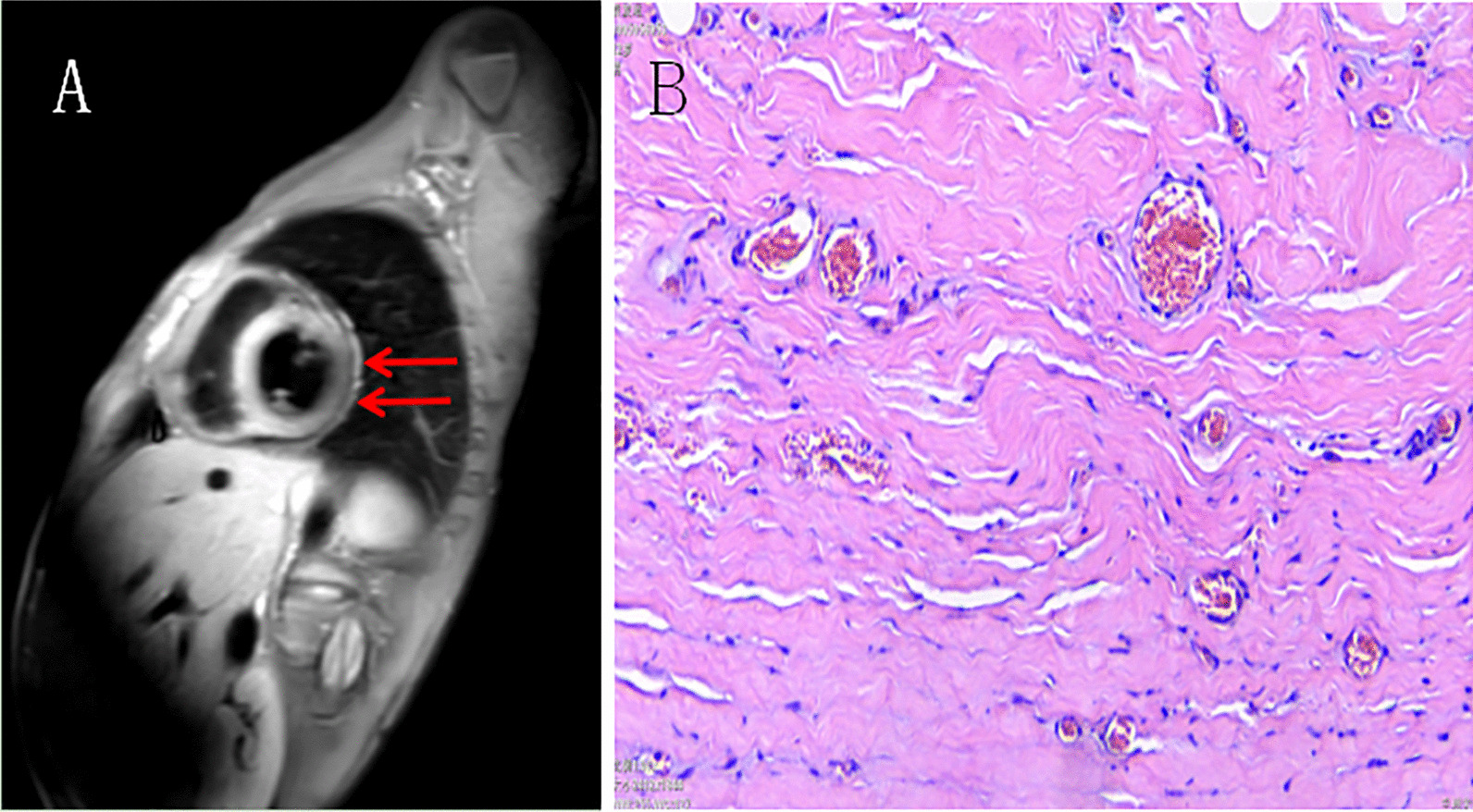
**A** MR showed abnormal signal changes in the interventricular septum and the middle parts of the anterior and inferior walls of the left ventricle. **B** The pathological demonstrated infiltration of chronic inflammatory cells in the stroma, vasodilation and hyperemia (H&E × 100)

After admission, ultrasound findings included partial pericardial thickening and adhesion (Fig. 1A); biatrial enlargement (especially the left atrium); obvious widening of superior and inferior vena cava, with decreased blood flow velocity, and disappearance of collapse rate of the inferior vena cava (indicating a significant increase in right atrial pressure); increased blood flow velocity of the mitral orifice with respiratory variability (Fig. 1B), with functional mitral regurgitation (moderate amount) and tricuspid regurgitation (small amount). The M pattern shows early diastolic septal jitter. Ultrasound suggested constrictive pericarditis (CP) with normal left and right ventricular function. Magnetic resonance imaging (MR) examination showed abnormal signal changes in the interventricular septum and the middle parts of the anterior and inferior walls of the left ventricle, which was mostly suspected as myocarditis (Fig. 2A). Resting SPECT for myocardial perfusion presented mild ischemic changes in the myocardium at the inferior wall of the left ventricle near the apex. Coronary angiography showed no obvious stenosis or plaque formation in the coronary artery. Laboratory results: In 2018, the patient was positive for IgM antibody to Epstein-Barr (EB) virus capsid antigen (EB-VCA-IgM) after acute infection with EB virus. Creatine kinase isoenzyme Mass (CKMB Mass) and high-sensitivity troponin T (hS-TNT) were increased. In 2018 and 2020, LgG antibody and LgM antibody of Coxsackie B virus (KSq IgG and KSq IgM) were negative. In 2020, the indicators of chronic infection of Epstein-Barr virus were positive for nuclear antigen IgG (EBNA1 IgG) and EB-VCA-IgG, positive for EB-VCA-IgM in acute infection, and significantly increased inβ-type natriuretic peptides, and T cell monitoring test of Mycobacterium tuberculosis infection was negative.

## Results

The clinical diagnosis was constrictive pericarditis with acute and chronic Epstein-Barr virus infection. In the Department of Cardiac Surgery, the child was treated with pericardiectomy for CP after anti-inflammatory therapy with proglobulin. Preoperatively, the central venous pressure was measured as about 18 cm Hg. Intraoperatively, obvious thickening of the pericardium was seen. After cutting open the pericardium, it was found that the thickness of the parietal pericardium was about 6 mm, with soft texture but adhesion to the heart. The postoperative recovery of the child was good. The pathological examination of the resected pericardium demonstrated a lot of necrotic tissues and proliferative collagen fibers, hyperplasia of granulation tissue, infiltration of chronic inflammatory cells in the stroma, vasodilation and hyperemia (Fig. 2B).The patient recovered well after surgery.

## Discussion/conclusion

CP is a disease with a serious limitation to cardiac diastolic filling resulted from pericardial thickening, adhesion and even calcification due to various causes, which often occurs in adults. At present, tuberculosis infection is still the main cause of CP in the world, accounting for about 4% [1]. In autopsy, the morbidity of CP in adults is about 1%, while that in children is unclear [2]. This child was diagnosed with CP caused by viral myocarditis. Firstly, this case was found in a child, and the incidence of CP is extremely low in children. Secondly, coxsackievirus is the most common cause of myocarditis [3]. Laboratory results showed that the patient had EB viral myocarditis and developed chronic myocarditis or pericarditis. Finally, the patient had no history of tuberculous bacterial infection, cardiac surgery and radiation treatment, and pericardial pathology showed chronic inflammatory changes. Consider chronic myocarditis leading to constrictive pericarditis, or myocarditis with pericarditis leading to constrictive pericarditis, which is rare. Because of long-term, chronic and repeated myocardial inflammatory cell infiltration, myocardial necrosis and inflammatory exudation involving the pericardium, pericardium thickening and adhesion occurred, resulting in a gradual limitation to ventricular filling, a significant increase in atrial pressure, cough, hepatomegaly and ascites. The onset of this child was slow and insidious, and laboratory and imaging examinations all suggested myocarditis, so it was not found until severe clinical symptoms appeared. At present, echocardiography is the main diagnostic method of this disease, but it is difficult to differentiate from restrictive cardiomyopathy, and requires high diagnostic level of sonographers. The main symptoms of the former include pericardial thickening, echo enhancement, atrial enlargement and more obvious changes in mitral E wave affected by respiration. In contrast, the latter showed whole-heart enlargement, and the e-wave rate of mitral valve was not affected by respiration. Tissue Doppler showed that the velocity of atrioventricular annulus motion significantly decreased in the early diastolic period (Fig. 1C). Two weeks before admission, echocardiography showed generalized cardiac enlargement, but no pericardial thickening. Retrospective analysis demonstrates that CP is rare in children, and there is no effusion in the pericardial cavity, the thickened pericardium resembles a fat pad, so it is easy to be missed. MRI has some limitations in the diagnosis of pericardial thickening, which is difficult to be differentiated from fat pad. In this case, there was no evidence of pericardial thickening on MRI in 2018 and this time. CT examination has X-ray exposure (especially in children) and poor repeatability. CT and MRI can’t dynamically observe the relative movement of the pericardium in real time, and the sedation time of infants and children is long during examination. However, color Doppler echocardiography is simple, flexible and repeatable, and CP has certain ultrasonic characteristics. Currently, pericardiectomy is the first choice for the treatment of CP [4, 5].

## Data Availability

Not applicable.
